# Screen for Cognitive Impairment in Psychiatry (SCIP): Adaptation and validation for Portuguese Version

**DOI:** 10.1192/j.eurpsy.2024.356

**Published:** 2024-08-27

**Authors:** B. Couto, R. Ortiga, A. S. Gonçalves, F. Monteleone, S. Oliveira, J. B. Fonseca, R. Rodrigues

**Affiliations:** ^1^Psychiatry, Hospital Senhora da Oliveira Guimarães, Guimarães, Portugal

## Abstract

**Introduction:**

Cognitive dysfunction has been reported in acute psychiatric patients for a long time and has profound implications for the management of severe mental disorders. The Screen for Cognitive Impairment in Psychiatry (SCIP) is a scale developed for screening cognitive deficits. This tool is simple and easy to administer.

**Objectives:**

To translate and to validate to Portuguese the SCIP.

**Methods:**

The accepted back-translation method is employed for translating from English into Portuguese. One-hundred individuals in good health were characterized using demographic questionnaires and a neuropsychological battery. Subsequently, the new version of the scale was administered on two distinct occasions with a minimum one-week gap between them.

**Results:**

High internal consistencies as well as strong correlations with comparable neuropsychological tests were obtained.

**Conclusions:**

The results obtained from the Portuguese version of SCIP are in line with those from the English version. Effectively, SCIP serves as a key instrument for the initial assessment of cognitive function. Its characteristics, particularly its conciseness and independence from a technological platform, allow it to be integrated into clinical practice. Our aim is to use this version and apply it to different pathologies, comparing patients with controls. This will allow us to study different patients and apply it to our population.

**Disclosure of Interest:**

None Declared

